# Factors Facilitating or Hindering Use of Evidence-Based Diabetes Interventions Among Local Health Departments

**DOI:** 10.1097/PHH.0000000000001094

**Published:** 2019-10-23

**Authors:** Allison R. Poehler, Renee G. Parks, Rachel G. Tabak, Elizabeth A. Baker, Ross C. Brownson

**Affiliations:** Prevention Research Center in St Louis, Brown School, Washington University in St Louis, St Louis, Missouri.

**Keywords:** chronic disease control, evidence-based programs and policies, local health departments, qualitative research

## Abstract

The objective of this study was to assess facilitators, barriers, and capacities to use of evidence-based programs and policies (EBPPs) in local health departments (LHDs).

**Design:**

A qualitative study design was used to elicit a contextual understanding of factors. One-hour interviews were conducted among directors and diabetes/chronic disease practitioners from LHDs. A consensus coding approach was used to identify themes.

**Setting and Participants:**

Twenty-four participants from 14 Missouri LHDs completed interviews.

**Results:**

Themes were identified as facilitators, barriers, or capacities that enhance EBPP use. Facilitators included awareness of EBPPs, leadership and supervisor support of EBPP use, and facilitators to increase capacity to implement EBPPs. Skills development, targeted messaging, and understanding of evidence-based decision-making (EBDM) terminology were needed. Barriers to EBPPs use were described at the individual, organizational, and interorganizational levels and included community buy-in, limited resources, relevance to partners, and time scarcity. Capacities included the ways LHDs learn about EBPPs, methods that influence the use of EBPPs, and resources needed to sustain EBPPs. Top ways to learn about EBPPs were in-person interactions. Staff meetings, meetings with decision makers, and relevant evidence influenced decision making. Resources needed were funding, organizational capacity, and partnerships. Directors' and practitioners' views differed on type of agency culture that promoted EBPP use, preferences for learning about EBPPs, ways to influence decisions, needs, and barriers to EBPPs.

**Conclusions:**

These findings can inform future strategies to support uptake of EBPPs in diabetes and chronic disease control in LHDs. LHDs have a good understanding of EBPPs, but subtle differences in perception of EBPPs and needs exist between directors and practitioners. Investment in capacity building and fostering an organizational culture supportive of EBDM were key implications for practice. By investing in employee skill development, LHDs may increase agency capacity. Researchers should use preferred channels and targeted messaging to disseminate findings.

Diabetes is the seventh leading cause of death in the United States, with more than 30 million adults living with diabetes.^1^ Moreover, 84.1 million adults have prediabetes, a condition associated with high blood sugar that increases risk of developing type 2 diabetes, heart disease, and stroke.^1^ The substantial impact of diabetes is unlikely to stagnate without dedicated control efforts. More than half of new diabetes cases are related to obesity, unhealthy eating, and physical inactivity.^2^,^3^ In the last 20 years, the number of adults diagnosed with diabetes has more than tripled.^1^

Although the burden of diabetes is great, it is largely preventable through application of evidence-based programs and policies (EBPPs), including diabetes self-management, obesity prevention, and development of registries to identify and control diabetes at a population level.^4^–^8^ Local health departments (LHDs) are well positioned to conduct diabetes EBPPs due to their knowledge of community needs, contexts, and resources, as well as their key role in the interface with health care providers and other community organizations.^9^ LHDs conduct public health activities including assessment of the problem, adaption of programs and policies, partnership building, and assuring EBPPs are effectively implemented.^10^–^13^ A quantitative study of LHDs in Missouri assessed the use of 20 diabetes-related EBPPs, feasibility of EBPPs, and personal/organizational barriers to evidence-based practice and found LHDs have an important role in implementing EBPPs.^14^ Findings indicate that more widespread adoption of EBPPs would be enhanced by increased education about and encouragement of evidence-based decision making (EBDM). EBDM involves making decisions based on the best available scientific or rigorous program evaluation evidence, applying program planning and quality improvement frameworks, engaging the community in assessment and decision making, adapting and implementing EBPPs for specific populations or settings, and conducting sound evaluation.^15^–^18^

A gap exists between availability of diabetes control EBPPs and actual diabetes control activities conducted by LHDs.^19^ Making EBPPs available for implementation in LHDs is not enough to assure widespread use.^20^–^22^ Local public health practitioners are aware of EBPPs but lack skills needed to adapt or use them. Organizational barriers also impede use.^15^,^23^–^26^ This qualitative study builds on our previous work exploring facilitators and barriers to implementation of EBPPs in the LHD context and utilizes key informant interviews conducted as part of a needs assessment in Missouri to prioritize and tailor approaches to diabetes control to local settings.^27^ The objective of this study was to understand various facilitators, barriers, and capacities of LHD directors and diabetes/chronic disease practitioners to implement and sustain EBPPs for diabetes control.

## Methods

A qualitative study design was utilized, as this approach allows for deeper probing and understanding of organizational, interorganization, and contextual factors that influence EBPP use. A convenience sample from already established LHD contacts with snowball sampling was employed to recruit participants from among the 115 LHDs in Missouri.

The project manager and 2 graduate research assistants conducted key informant interviews of local health directors or deputy directors and lead diabetes or chronic disease control practitioners in Missouri between January and April 2017. Interviews averaged 54 minutes in length and were conducted until saturation was reached, resulting in a total of 24. All interviews were administered by phone and audio recorded, transcribed verbatim following the interviews, and reviewed for completeness and accuracy. Participants were questioned on their awareness of the existence of diabetes control EBPPs, agency leadership and direct supervisor support for EBPPs, potential dissemination strategies and networks, how decisions about EBPPs are made or influenced within their agency, capacity-building needs for enhanced use of EBPPs, and EBPP implementation and sustainability supports. Ethical approval was obtained from the institutional review board of Washington University in St Louis (reference number: 201705026).

A consensus coding approach by 2 coders was used for analysis. This coding approach included segmentation of text using the interview guide to establish major categories, codebook development, coding, assessment of reliability, and iterative codebook modification as appropriate. Themes were identified, and director and practitioner responses were compared. A summary report including a list of potential dissemination activities was developed and shared with the study advisory committee. Data were managed and analyzed using NVivo 10 software.

## Results

### Sample characteristics

Twenty-four participants from 14 LHDs completed interviews. Participants who held LHD director (11) or deputy/assistant director (3) positions were considered directors. The 10 practitioners in diabetes or chronic disease control held various positions: 3 health educators, 2 public health nurses, 1 registered dietitian, 1 epidemiologist, and 3 program or division managers. Depending on the LHD size, practitioners may have been the only employee at their LHD in diabetes control or were the manager of a health promotion/chronic disease prevention program or division with several staff reporting to them. Eleven participants represented LHDs with small jurisdictions (<50 000 people), 7 represented LHDs with medium jurisdictions (50 000-199 999), and 6 participants were from LHDs with large jurisdictions (≥200 000). The majority of participants achieved a bachelor's degree (n = 8) or a master's degree in a field other than public health (n = 8). Six participants had a master's degree in public health. The majority of participants spent 11 years or greater working in public health.

### Theme overview

Overarching themes were facilitators, barriers, or capacities of LHDs to use and sustain EBPPs. Within these themes, individual, organizational, and interorganizational levels are described.

### Facilitators

#### LHDs are aware of EBPPs

Awareness of EBPPs for diabetes control is a crucial facilitator to using EBPPs for individual LHD employees and the organization as a whole. All practitioners were aware of EBPPs for diabetes control. Among directors, 3 indicated uncertainty as to whether a program, policy, or practice their agency implemented met the standards/criteria of evidence-based and 2 had no awareness of the existence of EBPPs. However, directors indicating uncertainty provided at least one example of an EBPP addressing diabetes risk factors implemented by their LHD during the past 12 months. One director elaborated on their hesitation to name an EBPP:

... I don't get to devote as much time to the research as I know that I should ... And when I look at some of the strategies that they [researchers] use, or their designs, there are some challenges with translating it ... and really make it evidence-based ...

Examples of EBPPs provided by respondents are listed in the Table.

**TABLE:** Examples of Diabetes and Chronic Disease Prevention and Control EBPPs Conducted in LHDs by Key Informants^a^

Primary Prevention Examples (n)	Secondary Prevention Examples (n)
Increasing PA in early child care centers (2) Increasing access to healthy foods in convenience and grocery stores (2) Smoke-free housing policy (1) Access to play spaces (1) Farm to school and school gardens (1) Adoption of land use and development with health considerations (1) Workplace wellness (1) Healthy lifestyle initiative for youth in primary care settings (campaign, healthy lifestyle assessment, and plan) (2) Breastfeeding promotion and support (spaces and policies) (1) Wellness programming (healthy eating and PA promotion) in church/faith community (2)	BP screenings and referrals to community health workers (1) Multicomponent coaching/counseling interventions to reduce weight (2) Diabetes screenings and education, including individualized counseling and support groups (2) Chronic disease self-management programs (3) Diabetes self-management programs (2) Diabetes and BP screenings at local food bank coupled with providing fresh veggies, fruits, and dairy products (1)

Abbreviations: BP, blood pressure; EBPPs, evidence-based programs and policies; LHDs, local health departments; PA, physical activity.

^a^The prevention examples listed here represent a range of scientific evidence supporting their effectiveness and were provided by the study respondents.

#### Leadership supports use of EBPPs for diabetes control

Another important facilitator to EBPP use among LHD employees and the organization is leadership support. Participants identified leaders in their agency as the health department board of directors, the medical or health officer, the director of the LHD, and division directors or managers within the LHD. All participants agreed leaders in their agency supported the use of evidence-based diabetes control.

Participants were asked about the type of support they received. The top 3 cited supports were: the LHD culture was supportive of new ideas and approaches, the culture was supportive of EBDM, and diabetes or chronic disease control is considered an agency priority and community need.

Directors reported their agency culture was supportive of new ideas and approaches as well as considered diabetes control an agency priority and community need. Directors also noted working across disciplines and utilizing software from other disciplines to streamline data collection and reporting.

Practitioners also noted supportive agency culture; however, they principally reported their cultures supported EBDM as compared with directors who commonly cited it is supportive of innovation. Practitioners noted expectations to use EBPPs and that using EBPPs is considered normative. One practitioner commented, “So I would say from the top down there's just the expectation, again, of this [EBDM] is how we're going to do business.” Specific support included tools, resources, and methodology for practitioners to develop evidence-based efforts and accountability to leadership and community to use evidence-based processes.

In addition to agency leadership support, practitioners were also asked about support they receive from their direct supervisors. All practitioners indicated they receive support from their direct supervisors for taking an evidence-based approach for diabetes control. In some cases, direct supervisors were also directors. Supports discussed most frequently were encouragement and recognition from supervisors, navigating partnerships, and strategizing and problem-solving assistance. One practitioner described the encouragement and recognition received from a supervisor:

When we do something that's ... really working in our community that was an evidence-based program, she goes to the effort to say, hey, thanks for doing that, you know, that's a good program.

#### Increasing LHD capacity

Participants discussed facilitators to increase organizational capacity to use and sustain EBPPs. Among all participants, the most reported ways to increase capacity were staff skills development, adequate funds, adequate staff, receiving targeted messages, a shared understanding of terminology used in EBDM among employees, and access to professional development trainings/resources. Differences in needs reported by directors and practitioners were reported (Figure).

**FIGURE:**
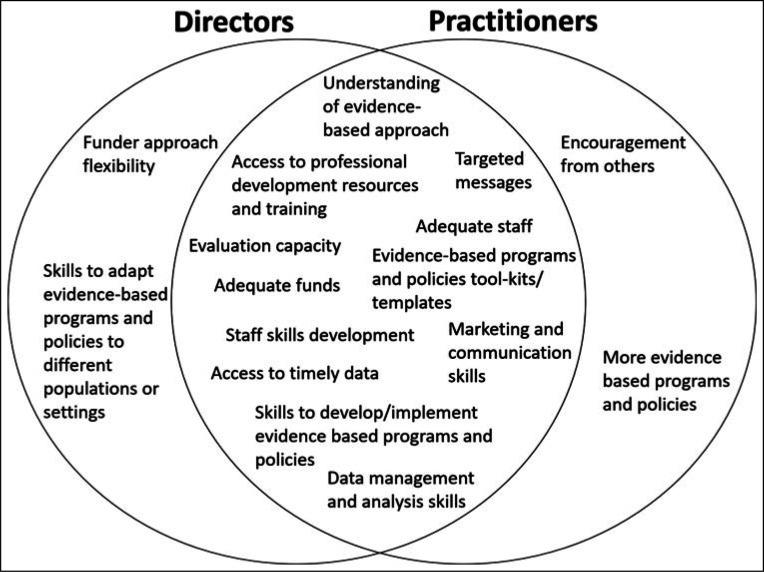
Directors and Practitioners Reported Agency Capacity Needs to Use EBPPs

Directors and practitioners in particular noted targeted messages, specifically, emails that contain current findings on EBPPs, supportive resources, and potential actions to take as a result of the findings as a way to increase use of EBPPs:

... We get so bombarded with emails and everything else, that it's hard to just keep up ... So if there was something that came in on a regular basis ... that consolidated or grouped information together so that you could learn all at one place without having to go to NACCHO, CDC, the Community Guide, and find it on your own, that'd be great.

When staff skills were noted as a need, interviewees were asked to provide specific examples. The most frequently cited skills in rank order were marketing and communication, data management and analysis, EBPP development and implementation, and adapting EBPPs to different populations and settings. Practitioners especially expressed needing marketing and communication skills to enhance messaging, mode, and reach:

A lot of times we have these evidence-based programs, but you kind of have to be your own marketing company to promote those programs. Not coming from a marketing or PR background, that is sometimes interesting to navigate. I think even a training or technical assistance on how to ... I guess training and ideas on how to really get that out there ...

#### Program capacity and partnerships sustain EBPPs

All participants listed funding, program capacity, and partnerships as top ways to implement and sustain EBPPs. Program capacity was characterized using the Center for Public Health Systems Sciences Program Sustainability Framework.^28^ It included having staff with relevant experience or training and resources to develop and retain competent staff, building partnerships, developing and implementing policy, and acquiring or maintaining needed program equipment. Directors more often cited program capacity as a support than practitioners. Practitioners more often cited partnerships as an essential ingredient for sustaining EBPPs:

*Well*, if you look at our community health plan, if we lost our core group that's driving the Community Health Improvement Plan, suddenly that whole thing would look pretty pathetic and we wouldn't accomplish a quarter of it.

### Barriers

After discussing facilitators to EBPP use, participants were questioned on organizational, individual, and interorganizational barriers to using EBPPs. Top organizational barriers for EBPPs were community perception or buy-in and limited resources including funding and number of staff. One director described how community perception of the agency became a barrier to implementing EBPPs when long-term programs ended, resulting in layoffs in a small community. Community perception was also described as a barrier when community members lacked awareness of diabetes risk factors and were not engaged in the decision-making process for EBPPs. Some participants noted their populations face financial instability and thus may be indifferent to efforts at diabetes control. Participants also noted EBPPs are not always accepted by the target population or there is a disconnect between the LHD and the target audience:

If you look at our county and you compare us to the rest of Missouri, we look pretty darn healthy. And so it's really hard for people ... to see that there is a need for additional resources around diabetes or chronic disease prevention. But if you actually dive into it a little bit deeper and you actually look at it by race, you see tremendous, tremendous disparities ...

Time was cited as a top individual barrier among both directors and practitioners:

... probably not enough time for quiet study and reflection, and learning ... I feel like over all these years, my ability to stay on top of all the things I want to know is dropping because of the other demands.

A top individual barrier among practitioners was feeling as if they were not an expert on relevant issues. One practitioner stated, “I'm always afraid they're going to ask a question that I can't answer, um, simply because I don't have that training, you know.”

Participants were also asked to describe barriers to building and maintaining interorganizational partnerships. The top barriers cited by all participants were relevance of the issue to the partner or the community and not enough time to connect and maintain relationships. Directors noted relevance of the issue or need to the partner or community as a barrier to increasing collaboration:

Sometimes the barrier could be people [at organization/agency] not identifying a need for a particular service or, if the problem even is there. Sometimes people just don't recognize that there is a need.

Practitioners also named time as a significant barrier to increasing collaboration compared with directors:

Everybody's trying to do more with less and it's whether or not you attend this meeting ... and I think a lot of times people are so busy with their day-to-day work that they're incapable of taking a step back and figuring out what really needs to be done. So time is a major barrier.

### Capacities of LHDs

#### Ways of learning about EBPPs

The top ways participants learned about EBPPs and new findings in rank-order were in-person conferences/seminars/meetings, professional associations, newsletters/email alerts, peers, and trusted websites. Local conferences and meetings were frequently mentioned by both directors and practitioners. One director described learning about EBPPs at grantee meetings:

... they would convene us into these grantee meetings, and so much learning happens there as you're sharing with your colleagues across the state about how they're approaching these initiatives ...

National associations like the American Public Health Association and the National Association of County and City Health Officials (NACCHO) as well as local professional organizations were additional resources for learning about EBPPs. The Centers for Disease Control and Prevention (CDC) was named as a trusted website along with the Community Guide, What Works for Health, Gallup, Robert Wood Johnson Foundation, and the NACCHO. Many participants also highlighted the role of peer interaction when learning about EBPPs.

More directors than practitioners reported professional associations as a way to learn about current findings in EBPPs. Most practitioners indicated webinars as a top method to learn about EBPPs whereas only one director noted webinars as a top method. Overall, this information provides an understanding of how LHDs prefer to receive information regarding EBPPs.

#### Methods of influencing use of EBPPs

Participants discussed methods they use to influence decisions regarding use of EBPPs within their agency and with partnering organizations. The top methods for making and influencing decisions on EBPPs among all participants were regular staff communications and meetings, meetings with internal and community decision makers, and obtaining and providing evidence relevant to the community. One director described how meetings with community decision makers were instrumental to using EBPPs:

We actually had executive level people in our community sitting around the table once a quarter talking about these issues ... they've signed on to this Community Health Improvement Plan and they help guide us through this process.

Most practitioners cited obtaining and providing evidence relevant to the community or audience as most useful when influencing decisions about EBPPs:

The thing that we try to do a lot in our community is ... “what's that mean here?” ... working with local data sets ... to provide that local perspective ... to see it through that lens, and how to support it with other trusted sources.

However, most directors noted regular staff communications and meetings as the most useful way to influence decisions about EBPPs.

## Discussion and Conclusion

The interviews provided insightful findings on similarities and differences among local health directors and practitioners on supports for EBDM, ways they learn about current findings in EBPPs, and capacity needs to further support use of evidence-based diabetes control. These findings support previous work on evidence-based practice in LHDs as well as provide a deeper understanding and context of facilitators and barriers to EBPP use. Overall, participants possess a good understanding of EBPPs but noted needing skill development and resources to evaluate and adopt EBPPs in their settings. Directors and practitioners differed on their views of how their organization supports EBPP use for diabetes control. Directors viewed their agency culture as fostering innovative approaches to support EBPPs use, while practitioners considered using EBPPs as a common practice. Even within the same LHD, a director used language like “valuing entrepreneurial thinking” and the practitioner simply stated using EBPPs was expected, thus resources were provided. This difference may be attributed to how directors and practitioners engage with EBPPs and frequency of engagement.

Practitioners noted receiving support to use EBPPs from leadership and their supervisors, indicating the importance of encouragement as a support for using EBPPs. However, some leaders were uncertain whether their programs were rooted in evidence or unaware of EBPPs for diabetes control, illustrating room for improvement. In addition, many practitioners emphasized the need for support at the individual and organizational levels when using and disseminating EBPPs in their prevention context. This finding supports previous work by Zwald and colleagues^14^ indicating encouragement from leaders as a high-priority practice within LHDs conducting EBPPs. Providing encouragement to practitioners is a simple and modifiable practice that health departments can utilize to support EBPPs.^32^

The interviews probed how information on EBPPs is disseminated to LHDs as well as how they use information to influence decision making, especially the emphasis from practitioners on using local data to influence decisions on EBPPs. Participants noted peer interactions, especially in the form of in-person conferences, seminars, and meetings, were the top way they learn about EBPPs. This supports previous findings that LHDs learn about academic findings through professional associations, seminars, and email alerts, and highlights the disconnect between how LHDs receive findings and how researchers communicate findings (ie, via journal articles).^33^ When influencing decisions on EBPPs, directors noted the importance of having community leaders who serve as decision makers as partners. Practitioners revealed they use local data to influence decisions on EBPPs.

All participants noted needing skill development to increase agency use of EBDM and EBPPs. Many of these competencies can be addressed via online or in-person training programs.^33^ Marketing and communication skills were the most discussed needed skill. Communication needs were expressed often throughout the interviews, with participants noting community buy-in as a barrier to using EBPPs as well as the need to utilize information relevant to communities. Participants also wanted skill development in implementing and adapting EBPPs to their settings. In addition to skill development, participants indicated adequate funds as an agency need to use EBPPs, as well as targeted messages. Participants noted email alerts as a common method of receiving information on EBPPs, but specifically expressed needing targeted and tailored messages that summarize EBPP findings, resources, and provide actionable steps LHDs can take to adopt EBPPs (as used in Dobbins et al).^34^ Targeted messaging is a strategy demonstrated to be effective at disseminating research evidence into public health programs and policies.^34^

LHDs are well poised to conduct diabetes and chronic disease prevention and control. Although gaps between research and practice exist, participants were well aware of EBPPs. However, specific LHD needs should be considered to enhance capacity to use EBPPs. The differences in perceptions between directors and practitioners should be considered when communicating new EBPPs and addressing capacity-building needs.

**Implications for Policy & Practice**

- These findings can inform future strategies to support uptake of evidence-based diabetes control among local-level practitioners and directors.
- Implications for LHD climate and culture include a need for agency leadership to foster an organizational culture supportive of EBDM—and with that a shared understanding of terminology or common language for EBDM among employees, management to provide encouragement to frontline public health workers to use EBPPs and recognition for EBPP implementation and impact, support for employees' awareness and learning about EBPPs through attendance at conferences/seminars/meetings, and access to resources for EBPPs and EBDM (from trusted website resources and professional associations).
- Investments in employee skill development through trainings covering a variety of topics listed may offset practitioners' perceived lack of expertise on relevant issues and increase agency capacity.^29^
- When investing in capacity building, LHDs are likely to benefit by skill enhancement in several key areas including identifying and adapting interventions, evaluation, and communicating research to policy makers.^30^
- LHD employees receive information on research findings from conferences, peers, national and local public health associations, and trusted websites. Researchers should use these preferred channels when disseminating findings to practitioners.^31^
- Webinars and targeted messages should be utilized when communicating diabetes and chronic disease control information to practitioners.
